# Identification of genetic variations linked to buparvaquone resistance in *Theileria annulata* infecting dairy cattle in India

**DOI:** 10.1371/journal.pone.0326243

**Published:** 2025-07-18

**Authors:** Pankaj Musale, Ajinkya Khilari, Rohini Gade, Velu Dhanikachalam, Santoshkumar Jadhav, Manali Bajpai, Bhagya Turakani, Akshay Joshi, Amar Prajapati, Anand Srivastava, Marimuthu Swaminathan, Sachin Joshi, Dhanasekaran Shanmugam

**Affiliations:** 1 Biochemical Sciences Division, CSIR- National Chemical Laboratory, Pune, Maharashtra, India; 2 Animal Breeding and Genetics Department, BAIF Development Research Foundation, Uruli Kanchan, Pune, Maharashtra, India; 3 Academy of Scientific and Innovative Research (AcSIR), Ghaziabad, India; 4 National Institute of Animal Biotechnology (NIAB), Hyderabad, Telangana, India; 5 Regional Centre for Biotechnology (RCB), Faridabad, Haryana, India; 6 Freelance Author; Guru Angad Dev Veterinary and Animal Sciences University (GADVASU), INDIA

## Abstract

Buparvaquone (BPQ) is used for the treatment of bovine theileriosis, a tickborne disease caused by parasites of the *Theileria* genus. Studies on *T. annulata* have linked the mechanism of BPQ resistance predominantly to genetic variations in the parasite cytochrome b (*cytb*) gene. In addition, cryptic mechanisms of resistance involving the parasite peptidyl-prolyl isomerase (*pin1*) and dihydroorotate dehydrogenase (*dhodh*) genes require assessment. In India, where bovine theileriosis is endemic, and BPQ is widely used for treatment, it is necessary to establish the prevalence of genetic variations linked to BPQ resistance. In this study, multiplexed PCR amplification and nanopore sequencing method was used for genotyping the complete gene loci of the three target genes. Analysis of 454 *T. annulata* field samples collected from seven different states of India revealed the presence of previously reported BPQ resistance associated variations S129G, A146T and P253S in *cytb* gene and A53P in *pin1* gene. The A146T and I203V variations in *cytb* were found to be prevalent and mostly co-occurring, and their role in BPQ resistance needs further evaluation. This study has revealed the presence of previously reported BPQ resistance-linked mutations in *cytb* and *pin1* genes in *T. annulata* infecting dairy cattle in India and establishes an Oxford nanopore sequencing method suitable for large-scale surveillance of genetic variation in *Theileria* parasites from field samples.

## Introduction

Bovine theileriosis in cattle is caused by parasites of *Theileria* genus [1], which are transmitted through the bites of infected ticks of three distinct genera of the Ixodidae family (hard ticks) [2]. *Theileria* species prevalence in different regions corresponds to the presence of specific tick vectors [3–5]. For example, *T. parva* (East Coast fever) is prevalent in the African continent, while *T. orientalis* (oriental theileriosis) is distributed more widely in Asia-Pacific, South Asia, Oceania, East Asia, India, and parts of Africa. *T. annulata* (tropical theileriosis) infections are more prevalent in North Africa, the Mediterranean coastal area, the Middle East, Central Asia, India, and Southeast Asia [6–8]. The clinical symptoms associated with theileriosis include heighten morbidity and can significantly undermine dairy productivity. In India, which has the largest cattle population globally, economic losses associated with theileriosis is estimated at more than US$ 780 million [9]. Disease surveillance and comprehensive control strategies, such as control of tick vectors using acaricides, vaccination of cattle (available in India as RAKSHAVAC-T), and treatment with drugs, have been employed to mitigate the impact of theileriosis [10–12].

Buparvaquone (BPQ; PubChem CID 71768), a naphthoquinone compound structurally related to the potent antimalarial drug atovaquone (ATQ), is the most effective drug available for theileriosis treatment and has been in use from the late 1980s [13]. Studies on the malaria parasite *Plasmodium falciparum* have provided mechanistic insights on how ATQ acts by targeting the CYTb protein which is a vital component of the mitochondrial respiratory complex III involved in the oxidation of reduced ubiquinone [14]. Mutations in the *P. falciparum cytb* gene can affect binding of ATQ to CYTb protein and result in resistance [15]. In similar manner to ATQ, BPQ likely exerts its inhibitory action by binding to the ubiquinone binding pocket of parasite CTYb and inhibiting its oxidation, as revealed from ATQ-bound yeast *cytb* structure [16].

Clinical treatment failure in cattle with tropical theileriosis, likely due to BPQ resistance, was first reported in studies from Tunisia [17]. In a study from Iran, the *T. annulata cytb* (*Tacytb*) gene from parasites derived from cattle with BPQ treatment failure was found to have two non-synonymous mutations resulting in S109G and P233S variation in the protein [18]. In another study, five mutations were found in the *Tacytb* gene from drug-resistant parasite isolates which resulted in I114M, S129G, P253S, L262S, S347L amino acid variations [19]. In a study from Sudan, analysis of the BPQ binding regions in the *Tacytb* gene revealed three mutations resulting in A146T, S129G, V227M amino acid variations [20]. From laboratory cultured parasites exhibiting BPQ resistance, *Tacytb* gene mutations resulting in A129G, P253S and L262S variations occurring in the ubiquinone binding Q_01_ and Q_02_ pockets was identified [21]. *T. annulata* isolates derived from animals with history of repeated BPQ treatment failure and showing higher IC_50_ values for BPQ inhibition in culture were seen to have *Tacytb* mutations corresponding to M51L, V135A, A146T, V227M, P253S variations [22]. *T. annulata* parasites induced to acquire BPQ resistance in laboratory culture were found to have the M128I mutation, which is equivalent to the M133I mutation in *P. falciparum cytb* linked to ATQ resistance. Molecular modeling studies with *Tacytb* revealed that M128I mutation may alter BPQ binding [23]. A recent study from India has reported identifying the I203V and I219V mutations in *Tacytb*, but their role in BPQ resistance has not been established [24].

Pyrimidine biosynthesis is essential for survival of apicomplexan parasites and a key enzyme of this pathway, dihydroorotate dehydrogenas*e* (DHODH), is in the parasite mitochondrion and functionally coupled to the bc1 component of respiratory complex III [25]. Thus, inhibition of CYTb function will result in compromised DHODH function and loss of pyrimidine biosynthesis. ATQ, and other naphthoquinone compounds, can probably directly bind and inhibit the DHODH enzyme. In fact, inhibition of recombinant *Schistosoma mansoni* and *Babesia bovis* DHODH enzyme activity by ATQ has been demonstrated [26,27]. In *P. falciparum*, mutations in the *dhodh* gene can confer ATQ resistance by an unknown mechanism [28,29]. Whether equivalent mutations occur in the *Theileria dhodh* gene that can confer resistance to BPQ remains to be explored.

The peptidyl-prolyl cis-trans isomerase NIMA-interacting 1 (*pin1*) gene encodes a proline isomerase involved in regulating the structure and function of proteins modified by proline-directed phosphorylation [30]. The *T. annulata* PIN1 protein is secreted into the infected leucocyte to facilitate the oncogenic transformation of the host cell [31]. Studies on field isolates of *T. annulata* from Tunisia and Sudan have shown that the A53P mutation is linked to BPQ resistance [32]. It was observed that BPQ can directly inhibit PIN1 activity in a heterologous system [31]. So far, there are no reports of the A53P mutation occurring in the *pin1* gene in *Theileria* isolates from India. To obtain a comprehensive picture on the nature and prevalence of genetic variations in *Theileria cytb*, *dhodh* and *pin1* genes, we have analyzed the sequence of these genes from *T. annulata* parasites infecting dairy cattle in different states of India. A targeted amplicon sequencing method using Oxford Nanopore Technology (ONT) was developed for genotyping the complete gene loci of *cytb*, *dhodh* and *pin1* genes from *T. annulata*. Analysis of field samples collected from seven different states of India has revealed the presence of previously reported *Tacytb* and *Tapin1* mutations linked to BPQ resistance as well as other novel variations.

## Methods

### Ethical declaration

The study protocol was approved by the Institutional Animal Ethics Committee (IAEC) of BAIF Development Research Foundation, Pune, Maharashtra, India (V-11011(13)/13/2023-CPCSEA-DADF). All activities were carried out in compliance with the guidelines set forth by the Committee for Control and Supervision of Experiments on Animals (CPCSEA). For collection of field samples consent was taken from cattle owners.

### Sample collection and processing

Blood samples were obtained from a total of 1798 crossbreed and native cattle from seven states of India, namely Maharashtra, Odisha, Jharkhand, Bihar, Uttar Pradesh, Punjab, and Gujarat. Out of these 1798 samples, 347 are prospective samples collected from Maharashtra only, while the remaining are retrospective samples and were collected from different states between 2018 and 2021 as part of the Enhanced Genetic Project Phase-1 carried out by BAIF Development Research Foundation, Pune. Sample-specific metadata, including animal, owner, and geography information, was collected. For the prospective samples, details of disease history, buparvaquone drug treatment and vaccination for theileriosis were collected when available (S1 Appendix). Blood samples were drawn from the jugular vein into K2 EDTA containing BD Vacutainer Eclipse Blood Collection tubes (BD, Franklin, USA). Blood smears were made on glass slides and Giemsa stained for microscopic examination to identify *Theileria* parasites in the samples. A detailed step-by-step compilation of all molecular protocols used in this along with reagent details is available in the Protocols.io document [dx.doi.org/10.17504/protocols.io.4r3l29zk4v1y/v1] and an abbreviated version is given here.

DNA was extracted from 200 µl of blood samples using the MagNA Pure 96 automated DNA extraction system (Roche, Switzerland), using the reagents and protocol from the manufacturer. To facilitate proper identification and tracking, the blood and corresponding DNA samples were sequentially labeled from BTH001 to BTH2178 (S1 Appendix). The quality and quantity of the isolated DNA was assessed using the NanoDrop spectrophotometer (Thermo Fisher Scientific, Waltham, MA, USA) and the DNA samples were stored at −20°C until further analysis.

### PCR amplification of *Theileria* 18S rRNA gene

PCR amplification of *Theileria* 18S rRNA gene locus was used as confirmation for parasite infection. A previously reported primer pair was used to amplify the 18S rRNA gene from *Theileria* species [33]. Two copies of the 18S gene are encoded by each species of *Theileria* on different chromosomes, and as the two genes have identical sequences, both will be amplified by the PCR primers. The genomic location coordinates for the 18S rRNA gene isoforms from different *Theileria* species are given in S1 Fig A. The PCR primer sequence is Forward 5′-GTGAAACTGCGAATGGCTCATTAC-3′, and Reverse 5′-AAGTGATAAGGTTCACAAAACTTCCC-3′. In India only *T. annulata* and *T. orientalis* infections are reported and the expected size of the 18S amplicons from these two species is 1608 and 1695 bp. PCR reactions were set up in 25μl using GoTaq® DNA Polymerase (Promega, USA) and 30–50 ng of template DNA. The PCR included an initial denaturation step for 4 minutes at 94°C, followed by 35 cycles of 45 second denaturation at 94°C, 1 minute annealing at 57°C, and 2 minute extension at 72°C. A final extension step at 72°C was set for 5 minutes. Positive controls for PCR reactions consisted of DNA extracted from laboratory cultured *T. annulata* (Ana2014 isolate from Anantapur district, Andra Pradesh State, India [34]) and field isolated *T. orientalis* (isolate from Wayanad district, Kerala state, India [35]). The PCR products were resolved by electrophoresis on a 1% agarose gel and visualized by ethidium bromide staining (S1 Fig B).

### Multiplexed PCR amplification of *Theileria* species *cytb*, *dhodh* and *pin1* genes

The primer pairs for PCR amplification of the *cytb*, *dhodh* and *pin1* genes were designed based on the sequence of the corresponding gene loci in *T. annulata* (*Ta*) and *T. orientalis* (*To*). The sequences were obtained from either VEuPathDB (https://veupathdb.org [36]) or NCBI (https://www.ncbi.nlm.nih.gov [37]) sequence repositories. The gene IDs, forward and reverse primer sequences, and amplicon size are as follows: *Tacytb* (Tap370b08.q2ca38.03c; 5′-CGGCGTTCTTAACCCAACTCA-3′ and 5′-GCGGTTAATCTTTCCTATTCCTTACG-3’; 1443 bp), *Tadhodh* (TA11695; 5′-CTCGCAAATCAACCAAAATCGCA-3′ and 5′-GGCGGTCACATTATGGTCACAA-3′; 1647 bp), *Tapin1* (TA18945; 5′-CAGCCTATGTTCAGAAGTTCAAACG-3′ and 5′-GGCGCTGAGAATAAAAGTGAACG-3′; 1474 bp), *TocytB* from Fish Creek strain (MACJ_004198; 5′-CCTCCCGACGTTTTTAACCCAA-3′ and 5′-TAACTGGCCCTGTTCGGTATTG-3′; 1464 bp), *Todhodh* from Shintoku strain (TOT_020000157; 5′-GTCTGGAAGCCTGCGGATATTT-3′ and 5′-TTTCATGTGAGCTGCTCCGATC-3′; 1731 bp) and *Topin1* from Shintoku strain (TOT_010000107; 5′-GACTGAGAATAGTTACCTCGAGCAG-3′ and 5′-AACAAGTGTGACGAGTCTACGC-3′; 1535 bp). Primers were designed using PrimalScheme (https://primalscheme.com/) and Primer3 tools, and the approximate size of the amplicons was maintained at around 1500 bp to obtain comparable amplification for all three genes in multiplexed PCR and unbiased read depth in nanopore sequencing.

PCR amplification was standardized for individual genes using DNA extracted from laboratory-cultured *T. annulata* (Ana2014 [34]) and field isolate of *T. orientalis* (Kerala isolate [35]). The PCR reaction included 50 ng of genomic DNA, 12.5 µl of the Quantabio repliQa HiFi ToughMix® and 3.5 µl of 10 µM primer mix in a total volume of 25 µl. The PCR amplification conditions were initial denaturation at 98°C for 30 seconds, 35 cycles of 15 second denaturation at 98°C and annealing plus an extension step at 65°C for 5 minutes, followed by holding at 4°C. Under the same standardized conditions, multiplexed amplification of the three genes was achieved for the two parasite species.

### Sequencing of PCR amplicons using Oxford Nanopore Technology

The multiplexed PCR products were purified using the AMPure XP beads (Beckman Coulter, USA) by mixing with each sample in a 1:1 volumetric ratio and incubating at room temperature for 15 minutes to allow DNA binding with beads. Samples were then placed on a suitable magnetic stand to separate the beads bound to DNA from the supernatant. The beads were washed twice with 1 ml of freshly prepared 70% ethanol and dried. Nuclease-free water (15–20µl) was added to the beads, mixed properly using a pipette and incubated for 15 minutes at room temperature to separate DNA from the beads. The beads were then captured by placing the tubes in a magnetic stand, and the DNA eluate was transferred to a new tube. The qualitative and quantitative analysis of the purified DNA was carried out using the nanodrop instrument (Thermo Fischer, USA), and 400–500 ng of each PCR product was taken for barcoding using the rapid barcoding SQK-RBK114.96 kit from ONT following the manufacturer’s protocol. The barcoded DNA samples were pooled and purified using the bead purification procedure as given above and eluted in 15–20 µl of elution buffer (EB). About 1 µg of the barcoded sample (in 11 µl) was mixed with 1 µl of rapid adapter ligation (RAP F) reagent and incubated at 37⁰C for 15 minutes to attach the ONT sequencing adapter. Before ONT sequencing, the flow cells (R.9.4.1) were primed as per the manufacturer’s protocol. The adapter attached DNA sample was mixed with 37.5 µl of sequencing buffer and 25.5 µl of loading beads and loaded onto the flow cell for sequencing. The nanopore sequencing was carried out using the GridIon sequencer and monitored using the MinKNOW V22.12.5 software [https://nanoporetech.com/document/rapid-sequencing-gdna-barcoding-sqk-rbk114].

Sequencing was performed until each target amplicon reached a minimum depth of at least 50x; the nucleotide level sequencing depth was monitored in real-time using the *T. annulata* rampart program which is deposited in Git Hub

[https://github.com/PankajMusale1988/RAMPART_TannulataMultiplex_Gene_Cytb_Dhodh_Pin1

]. Although only the *T. annulata* samples were considered for this study, the amplification and sequencing of the three genes from the Kerala isolate was used as reference sequence for *T. orientalis* from India. The rampart program for *T. orientalis* gene sequencing is also deposited in Git Hub [https://github.com/PankajMusale1988/RAMPART_TorientalisMultiplex_Genes_Cytb_Dhodh_Pin1]. From raw sequence reads, high-fidelity base calling was performed using Guppy in super-accuracy (SUP) mode, followed by reference-guided assembly in accordance with the high-quality analytical standards as outlined in the ARTIC-nCoV-bioinformatics SOP v1.1.0 (https://artic.network/ncov-2019/ncov2019-bioinformatics-sop.html) protocol. A Customized python script AmpAssem [https://github.com/ajinkyakhilari/ampAssem] was used for carrying out reference-guided assembly of sequence reads using minimap2 [38] along with depth masking using Bedtools [39] and variant mapping using bcftools [40].

Variant calling and annotation of SNP and indels was carried out using a customized python script AmpVarPro [https://github.com/ajinkyakhilari/AmpVarPro] which incorporates the Clair3 [41] and SnpEff [42] programs. To ensure the reliability of variant calling, stringent quality filtering criteria were applied, including the use of a minimum quality score threshold (QUAL > 10) to retain only high-confidence variants. Strand bias filtering was also performed, requiring sequence reads from both strands. Additional filtering steps included the application of strict mapping quality (MQ > 30) and base quality (BQ > 10) thresholds to eliminate unreliable variant calls. A minimum allele frequency (AF) threshold of 0.2 (20%) was set to exclude potential sequencing artifacts and retain only biologically meaningful variants.

The sequence data generated in this study can be accessed from GenBank under the Bio-Project ID PRJNA1110975. *T. annulata cytb, dhodh*, and *pin1* gene sequences can be accessed from submission ID SUB14616780 (data for Ana2014 isolate and 454 field samples with Metadata ID 14616780). *T. orientalis* data can be accessed from submission ID SUB14615033 (data for Kerala isolate and 60 field samples with Metadata ID 14615033).

### Multiple sequence alignment and phylogenetic analysis of 18S rRNA gene sequences of *Theileria* species

Multiple sequence alignments for *Theileria* 18S rRNA gene sequences were generated using the MAFFT alignment method [43]. The evolutionary history was inferred using the Neighbor-Joining method in MEGA11 [44] and the most optimal tree was obtained from bootstrap test (1000 replicates). The evolutionary distances were computed using the Maximum Composite Likelihood method [45] and are given in units of the number of base substitutions per site. The tree is drawn to scale, with branch lengths in the same units as those of the evolutionary distances used to infer the phylogenetic tree. The tree was edited using the Interactive Tree of Life (iTOL) [46] program for visualization and annotation purposes. The 18S rRNA gene sequences for reference strains were taken from their respective genome data available in NCBI: *T. annulata* Ankara strain (NC_011129.2:1688436–1690044) (Taxon ID: 353154); *T. orientalis* Shintoku strain (NC_025260.1:907127–908742) (Taxon ID: 869250); *T. orientalis* Fish Creek strain (CP056065.2:968495–970102) (Taxon ID: 68886); *T. orientalis* Goon Nure strain (CP056069.2:987414–989023) (Taxon ID: 68886); *T. parva* Muguga strain (NC_007344.1:923036–924644) (Taxon ID: 333668); *T. equi* WA strain (NC_021366.1:2591983–2593598) (Taxon ID: 1537102). The *B. bovis* T2 Bo (L19077.1) (Taxon ID: 484906) and *P. falciparum* (XR_002273095.1) (Taxon ID: 36329) sequences were taken from GenBank. The 18S rRNA gene sequences generated in this study for the *T. annulata* Ana2014 isolate and the *T. orientalis* Kerala isolate were included to represent India genotypes of these two species.

## Results

### Sample collection and identification of *Theileria* infection by 18S rRNA gene amplification

For prospective sampling, blood samples were collected from cattle with history of tick infestation and/or suspected to have theileriosis based on clinical symptoms such as fever, swollen lymph node (prescapular), diarrhea and occasional pinpoint hemorrhages in the vaginal mucosa. Individual animal-associated metadata was also collected which included information on the animal breed, disease history, drug treatment and vaccination against theileriosis (S1 Appendix). Based on positive PCR amplification of the *Theileria* 18S rRNA gene fragment, 209 samples, from a total of 347 prospective samples, were identified with *Theileria* infection. Retrospective analysis was carried out on 1451 samples collected from seven different states of India. From positive amplification of 18S rRNA gene fragment, 810 samples were identified as *Theileria* infected. In total, 1019 18S rRNA gene PCR positive samples (209 prospective and 810 retrospective) were taken ahead for sequencing and genotyping the parasite *cytb*, *dhodh* and *pin1* genes. In addition, the sequence of these three genes were generated for the *T. annulata* Ana2014 and *T. orientalis* Kerala isolates and used as reference Indian genotypes. It was observed that the 18S rRNA gene sequences of the Ana2014 and Kerala isolates grouped within the *T. annulata* and *T. orientalis* clades in the phylogenetic tree (S1 Fig C). This confirmed that the primers used for 18S rRNA gene PCR can amplify the gene from both *T. annulata* and *T. orientalis* parasites present in field samples. However, *Theileria* species discrimination was achieved by *cytb*, *dhodh* and *pin1* gene amplification using species-specific PCR primers.

### Multiplexed amplification and sequencing of *Theileria cytb*, *dhodh* and *pin1* genes

**Fig 1:**
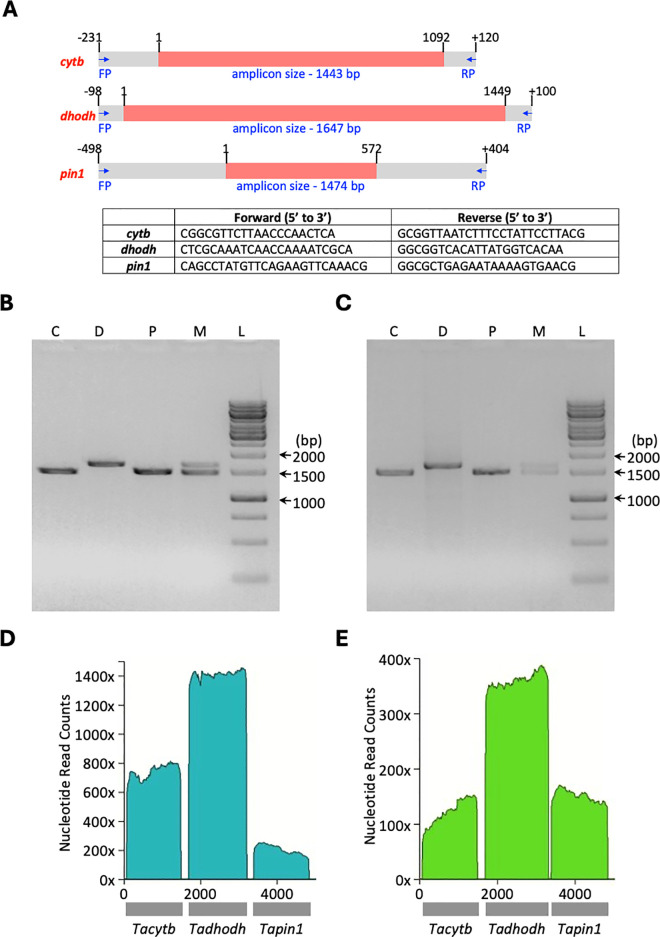
A, Schematic representation of coding region of *T. annulata cytb*, *dhodh*, and *pin1* genes (red colour) including flanking sequences (grey colour) is shown along with a table listing the PCR primer used in the study. PCR amplification (B, C) and nanopore sequencing (D, E) of *cytb* (C), *dhodh* (D), *pin1* (P) and all three genes multiplexed (M) from *T. annulata* species. DNA templates used for PCR and sequencing were obtained from Ana2014 strain (B, D) and a representative field sample (C, E). Lane L in B and C, 1 kb DNA marker ladder. Nucleotide-level mapping of nanopore sequence data for multiplexed PCR amplicons was plotted in real-time using the RAMPART program.

For genotyping the *cytb, dhodh and pin1* genes from field samples, primers were designed based on the reference gene sequences from *T. annulata* Ankara strain (Fig 1A). As some of the field samples are expected to have *T. orientalis* infection and to distinguish these samples from *T. annulata* samples, primers for amplification of *cytb, dhodh and pin1* genes were designed based on the reference gene sequences from *T. orientalis* Shintoku genotype (S2 Fig A). Initially, PCR conditions were established by individual amplification of the three genes, followed by multiplexed amplification of all three genes in a single PCR. The *T. annulata* Ana2014 isolate was used as a positive control (Fig 1B) to establish the PCR conditions which also worked well for the field sample (Fig 1C). The multiplexed PCR amplicons from the control and field samples were sequenced by nanopore technology; Fig 1D and 1E show the nucleotide level Rampart mapping of the real-time base calls from nanopore sequencing. For *T. orientali*s, the PCR amplification of the three genes was established using DNA obtained from the Kerala isolate and nanopore sequencing was carried out to obtain the respective gene sequences (S2 Figs B, C, D and S2E). Amplification for *T. orientalis* genes was done only in samples which were negative for PCR amplification of *T. annulata* genes.

Out of 1019 field samples positive for *Theileria* 18S rRNA gene PCR, 635 were identified as *T. annulata* infected samples and 98 samples were identified as *T. orientalis* infected samples, based on species-specific amplification of *cytb*, *dhodh* and *pin1* genes. For the remaining 286 samples the three genes could not be PCR amplified. Based on the quality of PCR amplicons, nanopore sequence data for the three genes was obtained from 454 *T. annulata* samples. Reference based variant calling was carried out with consensus sequence data generated with at least 60X `depth at the nucleotide level (Table 1).

**Table 1:** Summary of field samples collected and analyzed from different States of India.

Sample Type	State	Total number of samples collected	Samples positive for 18S rRNA gene PCR	Samples from which *T. annulata cytb, dhodh & pin1 *genes were sequenced
**Prospective**	Maharashtra	347	209	98
**Retrospective**	Maharashtra	237	146	36
	Gujarat	42	32	28
	Jharkhand	102	53	18
	Odisha	107	101	88
	Bihar	224	153	83
	Uttar Pradesh	283	188	50
	Punjab	456	137	53
**Total**		1798	1019	454

### Genetic variations in the *cytb* gene

**Fig 2:**
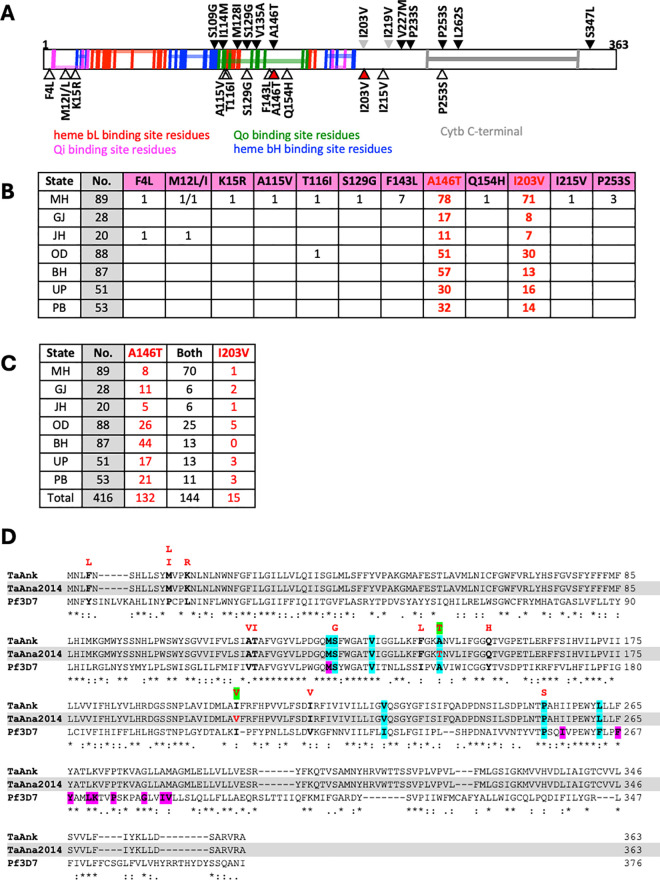
A, Schematic representation of *T. annulata* CYTb protein annotated with vertical-coloured lines indicating the conserved residues in functional domains shown by horizontal shading (details taken from conserved domain database. https://www.ncbi.nlm.nih.gov/Structure/cdd/cdd.shtml). The black-filled arrowheads shown at the top are the previously reported variations identified from parasites exhibiting resistance to BPQ. The grey-filled arrowheads represent the variations reported from India [24]. The open and red-filled arrowheads shown at the bottom are the variations identified in this study from field samples compared to the *T. annulata* reference sequence. The two highly prevalent and co-occurring variations A146T and I203V are highlighted by red-filled arrowheads. **B**, List of the number of field samples analyzed from each state (column 2; grey highlight) and number of samples with different *Tacytb* mutations. Data for the two most prevalent variations is shown in red. MH, Maharashtra; GJ, Gujarat; JH, Jharkhand; OD, Odisha; BH, Bihar; UP, Uttar Pradesh; PB, Punjab. **C**, Co-occurrence of A146T and I203V mutations in *Tacytb* gene. State-wise sample number is shown in column 2, and number of samples with either one or both mutations is given. State names are abbreviated as in B. D, Multiple sequence alignment of CYTb protein sequences of *T. annulata* Ankara reference strain (TaAnk), *T. annulata* Ana2014 Indian isolate (TaAna2014) and *P. falciparum* 3D7 strain (Pf3D7). Positions of the previously reported mutations linked to BPQ resistance in *T. annulata* are highlighted in cyan on all three sequences. The positions with variations detected in field samples are shown in bold black fonts and the variant residues observed in these positions are shown in red font above the alignment. The A146T and I203V variation is highlighted in green and the corresponding position in the TaAna2014 sequence is shown in red font. The purple highlighted residues in Pf3D7 *cytb* are mutated in parasites exhibiting atovaquone resistance.

Various *cytb* gene mutations causing BPQ resistance have been reported from *T. annulata* parasites [17–24]. These mutations occur within the ubiquinone binding region (Qo binding site) and in the C-terminal region of the protein (Fig 2A). Three of the previously reported mutations associated with BPQ resistance (S129G, A146T and P253S) were detected in field samples from India. In addition to these 3 variations, 10 other *cytb* variations were detected from field samples. Four variations were found within the first 15 amino acids of the protein, while the other 6 variations were found in the heme and ubiquinone binding sites (Fig 2A). The role of these new variations in BPQ resistance will have to be studied further. Except for 2 variations, A146T and I203V which mostly co-occurred, all others were found to occur in very low frequency (Figs 2B and 2C). Data for BPQ treatment, either alone or in combination with other drugs, was available for 23 prospective samples with *T. annulata* infection. In 17 out of 23 samples, A146T and I203V co-occurred, 3 samples had A146T only and 3 samples had no variation. A115V, Q154H and P253S variations were seen in one sample each and co-occurred with both A146T and I203V (S3 Fig). Failure of BPQ treatment to cure *Theileria* infection in these cattle suggests that A146T and I203V variations may have a role in BPQ resistance, but needs to be evaluated. It is also noteworthy that the *P. falciparum cytb* mutations linked to ATQ resistance are in the C-terminal region of the protein except for one mutation which occurs in the Qo binding site (Fig 2D) [15,47]. Thus, the binding site preference and resistance mechanism for the two drugs, which are structural analogs appear to be distinct.

### Genetic variations in the *dhodh* gene

**Fig 3:**
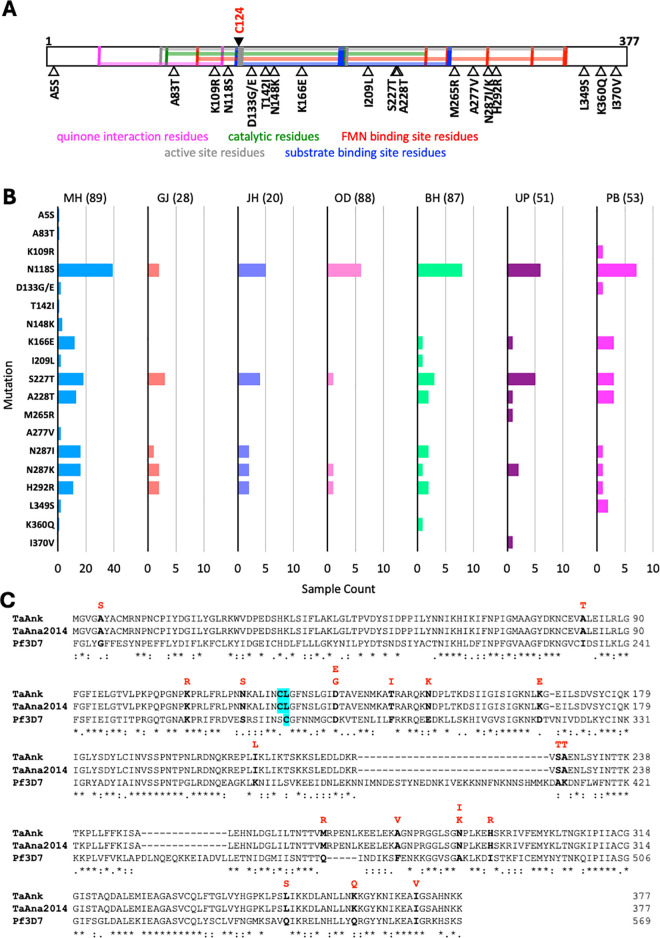
A, Schematic representation of *T. annulata* DHODH protein annotated with vertical-coloured lines indicating the conserved residues in functional domains shown by horizontal shading (details taken from conserved domain database. https://www.ncbi.nlm.nih.gov/Structure/cdd/cdd.shtml). The black-filled arrowhead indicates the C124 residue which corresponds to the C276F mutation in *P. falciparum dhodh* which confers atovaquone resistance. The open arrowheads shown at the bottom are the variations identified in this study. **B**, Prevalence of *T. annulata dhodh* mutations in field samples from different states of India (name of state abbreviated as in **Fig 2B**; the number of samples analyzed from each state is given within parentheses). **C**, Multiple sequence alignment of DHODH protein sequences of *T. annulata* Ankara reference strain (TaAnk), *T. annulata* Ana2014 Indian isolate (TaAna2014) and *P. falciparum* 3D7 strain (Pf3D7). The cyan highlighted residue in the Pf3D7 sequence indicates the C276 position which is mutated to confer atovaquone resistance. The corresponding residue in the *T. annulata* is a leucine (L125) and so the neighboring cysteine residue (C124) is considered; C124 and L125 are highlighted in cyan in TaAnk and TaAna2014 sequences. The residues shown in bold black font are altered in the field samples analyzed in this study. The variant residues observed in these positions are shown in red font above the alignment.

So far, there is no report linking genetic variation in the *dhodh* gene from *Theileria* species to BPQ resistance. In this study, the *dhodh* gene was sequenced from *T. annulata* to see if there is any genetic variation equivalent to the *P. falciparum* C276F mutation (Fig 3A). Gene sequencing will also help in identifying other genetic variations that are prevalent in the parasite population which can be further studied for its link to BPQ resistance. In *T. annulata* field samples, 20 different genetic variations were seen in the *dhodh* gene; 16 of these variations were present in the region containing the catalytic and flavin mono-nucleotide (FMN) binding residues (Fig 3A). Although the position corresponding to the *P. falciparum* C276F mutation [30] is a leucine (L125) in *T. annulata dhodh*, the neighbouring residue is a cysteine (C124). Both L125 and C124 residues were not altered in any of the field samples. The N118S variation was found to be the most prevalent (Fig 3B and 3C) and occurs in the vicinity of C124 residue, and therefore, its role in BPQ resistance needs to be evaluated.

### Genetic variations in the *pin1* gene

**Fig 4:**
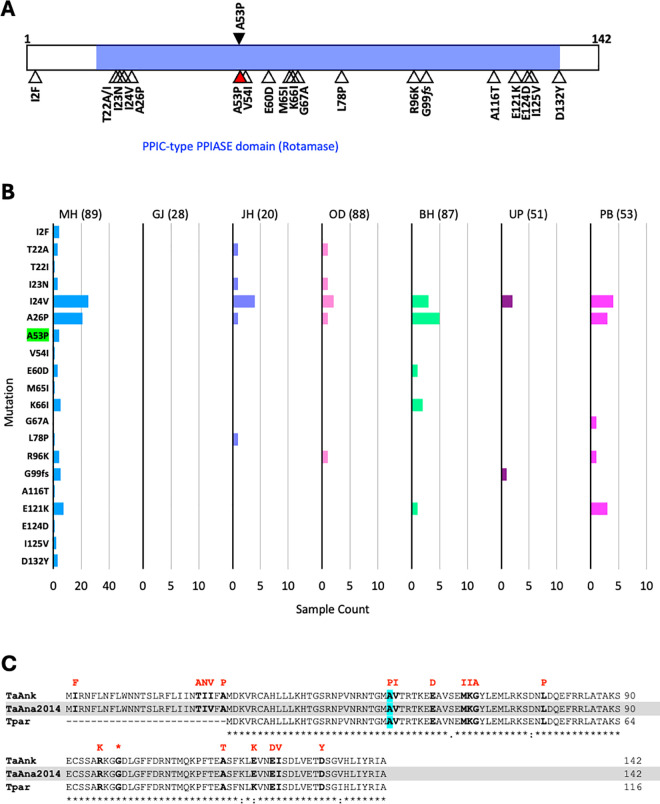
A, Schematic representation of *T. annulata* PIN1 protein annotated with functional domain shown by horizontal shading (details taken from conserved domain database. https://www.ncbi.nlm.nih.gov/Structure/cdd/cdd.shtml). The black-filled arrowhead shown at the top identifies the previously reported A53P variation linked to BPQ resistance. The open and red-filled arrowheads shown at the bottom are the variations identified from field sample analysis in this study. **B**, Prevalence of *T. annulata pin1* mutations in field samples from different states of India (name of state abbreviated as in Fig 4B; the number of samples analyzed from each state is given within parentheses). The A53P mutation identified from the field sample is highlighted in green. **C**, Multiple sequence alignment of PIN1 protein sequences of *T. annulata* Ankara reference strain (TaAnk), *T. annulata* Ana2014 Indian isolate (TaAna2014) and *T. parva* Muguga strain (Tpar). The A53P mutation is highlighted in cyan. The residues shown in bold black font are altered in the field samples analyzed in this study. The variant residues observed in these positions are shown in red font. A frameshift mutation detected at position G99 is indicated with * in red font.

The sequence of the *pin1* gene from *T. annulata* field samples revealed 19 variations, all of which were found in low frequency in the samples, except the I24V and A26P variations which were found in samples from all states except Gujarat (Figs 4A and 4B). The A53P mutation previously shown to confer BPQ resistance in cell culture studies [31] was also detected, but only in four samples from Maharashtra. In a field study from Sudan [32], one sample was found to have parasite with the A53P mutation. Although the A53P mutation is found in low frequency in our study and in the Sudanese study, its presence suggests a potential alternate mechanism for BPQ resistance. Hence the A53P mutation needs to be monitored in the parasite population and its effect on BPQ resistance evaluated further. The *pin1* gene is highly conserved between *T. annulata* and *T. parva* (both of which cause transforming theileriosis), and the variations detected in the field samples are predominantly in positions that are conserved in the two species (Fig 4C). Further studies are needed to ascertain the effect of the observed *pin1* variations on protein function or BPQ sensitivity.

## Discussion

Theileriosis is a prevalent disease in dairy cattle in India, responsible for losses in both dairy productivity and the economy [48,49]. Although tick control measures are available, a high disease transmission level exists, and cattle treatment with anti-parasitic agents is widely used [10–12]. The most effective drug available for the treatment of theileriosis is BPQ, but treatment failure has been reported due to drug resistance [17]. While BPQ resistance in *Theileria* parasites is predominantly due to genetic variations in *cytb* gene [18,19], there is evidence that a single mutation in the parasite *pin1* gene, A53P, is capable of conferring BPQ resistance [32]. While there is no evidence that mutations in *Theileria dhodh* gene can confer BPQ resistance, *dhodh* mutations in *P. falciparum* can confer ATQ resistance [28,29]. Recent studies in *P. falciparum* have reported that a very high level of resistance to the antimalarial drug ATQ is seen in parasites carrying the C276F mutation in the *dhodh* gene [50]. ATQ and BPQ are structural analogues, and act by binding to and inhibiting CYTb protein. Moreover, the activity of the DHODH enzyme is coupled to the redox activity of the CYTb protein present in the mitochondrial respiratory complex III of both *Plasmodium* and *Theileria* parasites. Due to these similarities, evaluating the genetic variation in the *dhodh* gene was of interest.

This study was carried out to assess the presence and prevalence of previously reported BPQ resistance-conferring mutations in *cytb* and *pin1* genes and to discover new genetic variations in *cytb*, *pin1* and *dhodh* genes in *Theileria* parasites infecting dairy cattle in India. PCR amplification and nanopore sequencing were carried out for genotyping the parasite *cytb, dhodh* and *pin1* genes from *T. annulata*. Out of 1798 field samples, 1019 were positive for *Theileria* infection based on 18S rRNA gene PCR. Based on the species-specific PCR amplification of *cytb*, *dhodh* and *pin1* genes, a majority of the samples were found to have *T. annulata* infection, which is the prevalent parasite in India [48].

Among the genetic variations identified from *T. annulata cytb* gene the A146T and I203V variations were the most prevalent. The A146T mutation, located in the Qo binding site of *cytb*, was originally reported to be prevalent in Sudan where it was found in all 50 *T. annulata* samples analysed [20]. The I203V variant, which was previously reported from India [24], was also detected in this study and was found to be widely prevalent and mostly co-occurring with A146T. Two other previously reported BPQ resistance-linked variations, S129G and P253S, which were identified and linked to BPQ resistance in multiple studies [19–21], were also detected but only from one field sample each. Despite their low prevalence, the presence of previously reported mutations linked to BPQ resistance suggests the potential for causing BPQ resistance in India. Genetic surveillance studies are needed to map the prevalence of these mutations in *T. annulata* in India and study their association to BPQ treatment failure.

In case of the *dhodh* gene, the L125 residue (same position as the C276 residue mutated in *P. falciparum dhodh* exhibiting ATQ resistance), and the neighbouring C124 residue did not show any variation. The A53P variation was detected in the *pin1* gene sequence of four field samples, suggesting that BPQ resistance mediated by *pin1* mutation might exist in India. In both *dhodh* and *pin1* genes*,* many other variations were detected in *T. annulata* field samples that must be evaluated in the context of BPQ resistance. Future studies in which BPQ treatment outcomes are correlated with *cytb*, *dhodh* and *pin1* genotypes are needed for assessing the role of genetic variations in these genes on BPQ resistance in *Theileria* species affecting dairy cattle in India.

## Conclusion

This work lays the foundation for cataloguing and understanding the genetic variations in *Theileria* parasite, specifically focusing on mutations in the *cytb, dhodh* and *pin1* genes that can be linked to BPQ resistance. The findings from this study reveal that *T. annulata* parasites from India harbour previously reported genetic variations linked to BPQ resistance. These findings emphasise the critical need for continuous monitoring of BPQ resistance-linked genetic variations. This study also establishes an amplicon sequencing method using Oxford Nanopore Technology, as a scalable method for surveillance studies on genetic variation in *Theileria* parasites affecting dairy cattle in India. Further research into the significance of the novel genetic variations identified in this study and their effect on BPQ drug efficacy is necessary to optimise control measures against this economically important parasitic disease.

## Supporting information

S1 Fig 18S rRNA gene PCR amplification and phylogeny. A, Table listing the NCBI accession details for the two 18S rRNA gene isoforms present in different *Theileria* species. The chromosome number (chr-1, chr-3 & chr-4), NCBI sequence ID and sequence coordinates are given for each gene isoform. B, Agarose gel electrophoresis of 18S rRNA gene PCR amplicons from *Theileria* parasites. Lane markings: B, no template control; 1, *T. annulata* Ana2014 isolate 18S amplicon; 2, *T. orientalis* Kerala isolate 18S amplicon; 3–5 & 7, field samples positive for *Theileria* 18S gene amplicons; 6, field sample negative for *Theileria* 18S gene amplicons; L, 1 kb DNA marker ladder. C, Phylogram of 18S rRNA gene sequences from reference strains of *Theileria species* and Indian isolates of *T. annulata* (Ana2014) and *T. orientalis* (Kerala). *Babesia bovis* and *Plasmodium falciparum* were included as outgroups.
(TIF)

S2 Fig PCR amplification and sequencing of *T. orientalis cytb*, *dhodh* and *pin1* genes. A, Schematic representation of coding region of *T. orientalis cytb*, *dhodh*, and *pin1* genes (red colour) and flanking sequences (grey colour) is shown along with a table listing the PCR primers used in the study. Representative data for PCR amplification (B, C) and nanopore sequencing (D, E) of *cytb* (C), *dhodh* (D), *pin1* (P) and multiplexed analysis of all three genes (M) from *T. orientalis* species. DNA templates used for PCR and sequencing were obtained from Kerala isolate (B, D) and a representative field sample (C, E). Lane L in B and C, 1 kb DNA marker ladder. Nucleotide-level mapping of nanopore sequence data for multiplexed PCR amplicons was plotted in real-time using the RAMPART program.
(TIF)

S3 Fig Details of *Tacytb* gene mutations identified in samples with BPQ treatment data. The genetic variations detected in the *Tacytb* gene from each sample is shown in red shading. The last column shows that drug treatment given for the individual animals.
(TIF)

S1 Appendix Sample metadata details. The sequence data generated from the samples have been deposited in GenBank under the Bio-Project ID PRJNA1110975 (see further details in methods section of manuscript). The sample IDs given in this sheet can be used to access sample specific details from the GenBank submission.
(XLSX)
