# Supplementary figures and images for: Identification of genetic variations linked to buparvaquone resistance in Theileria annulata infecting dairy cattle in India
Source: PLoS One. 2025 Jul 18;20(7):e0326243. doi: 10.1371/journal.pone.0326243 (PMC12273908; doi:10.1371/journal.pone.0326243)

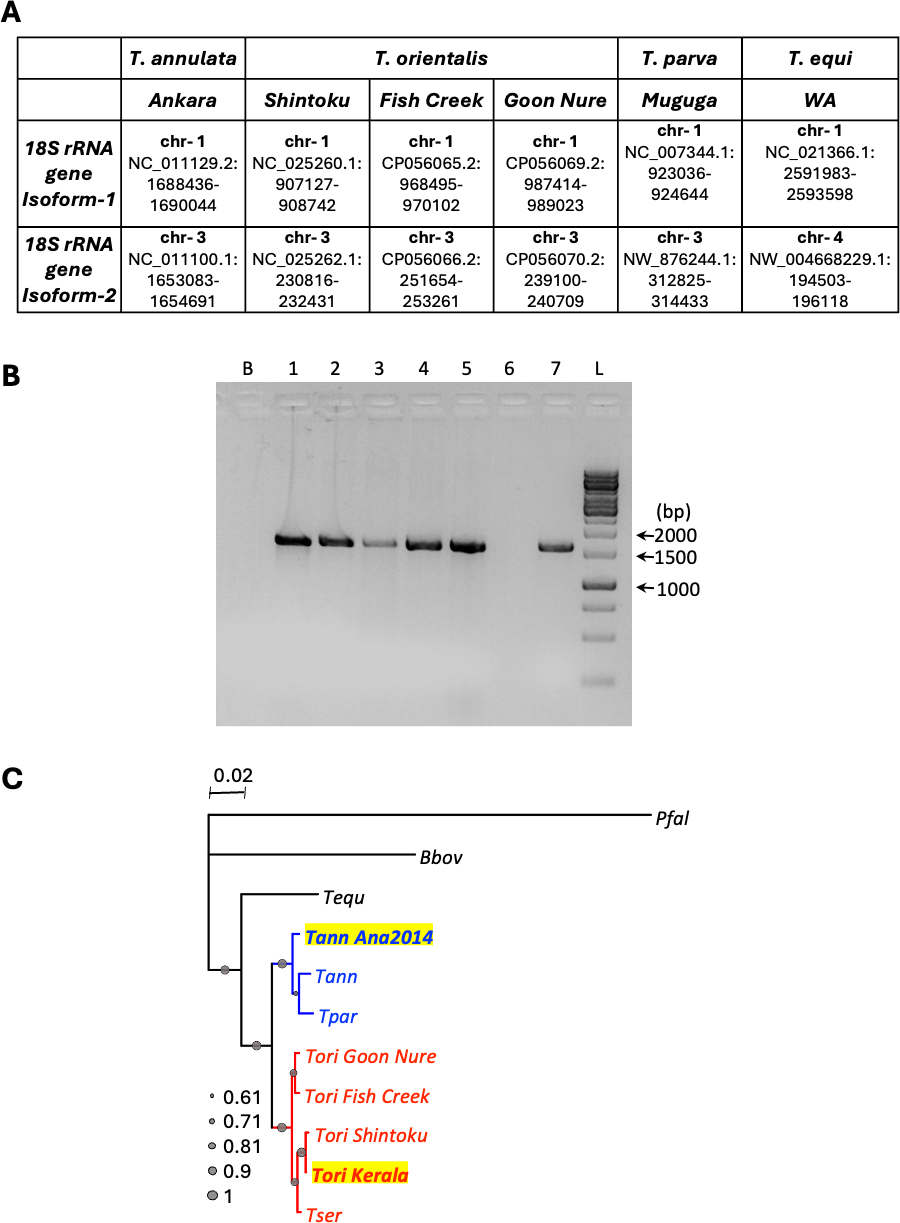

Supplement: S1 Fig — A, Table listing the NCBI accession details for the two 18S rRNA gene isoforms present in different Theileria species. The chromosome number (chr-1, chr-3 & chr-4), NCBI sequence ID and sequence coordinates are given for each gene isoform. B, Agarose gel electrophoresis of 18S rRNA gene PCR amplicons from Theileria parasites. Lane markings: B, no template control; 1, T. annulata Ana2014 isolate 18S amplicon; 2, T. orientalis Kerala isolate 18S amplicon; 3–5 & 7, field samples positive for Theileria 18S gene amplicons; 6, field sample negative for Theileria 18S gene amplicons; L, 1 kb DNA marker ladder. C, Phylogram of 18S rRNA gene sequences from reference strains of Theileria species and Indian isolates of T. annulata (Ana2014) and T. orientalis (Kerala). Babesia bovis and Plasmodium falciparum were included as outgroups. (TIF) [file pone.0326243.s001.tif]

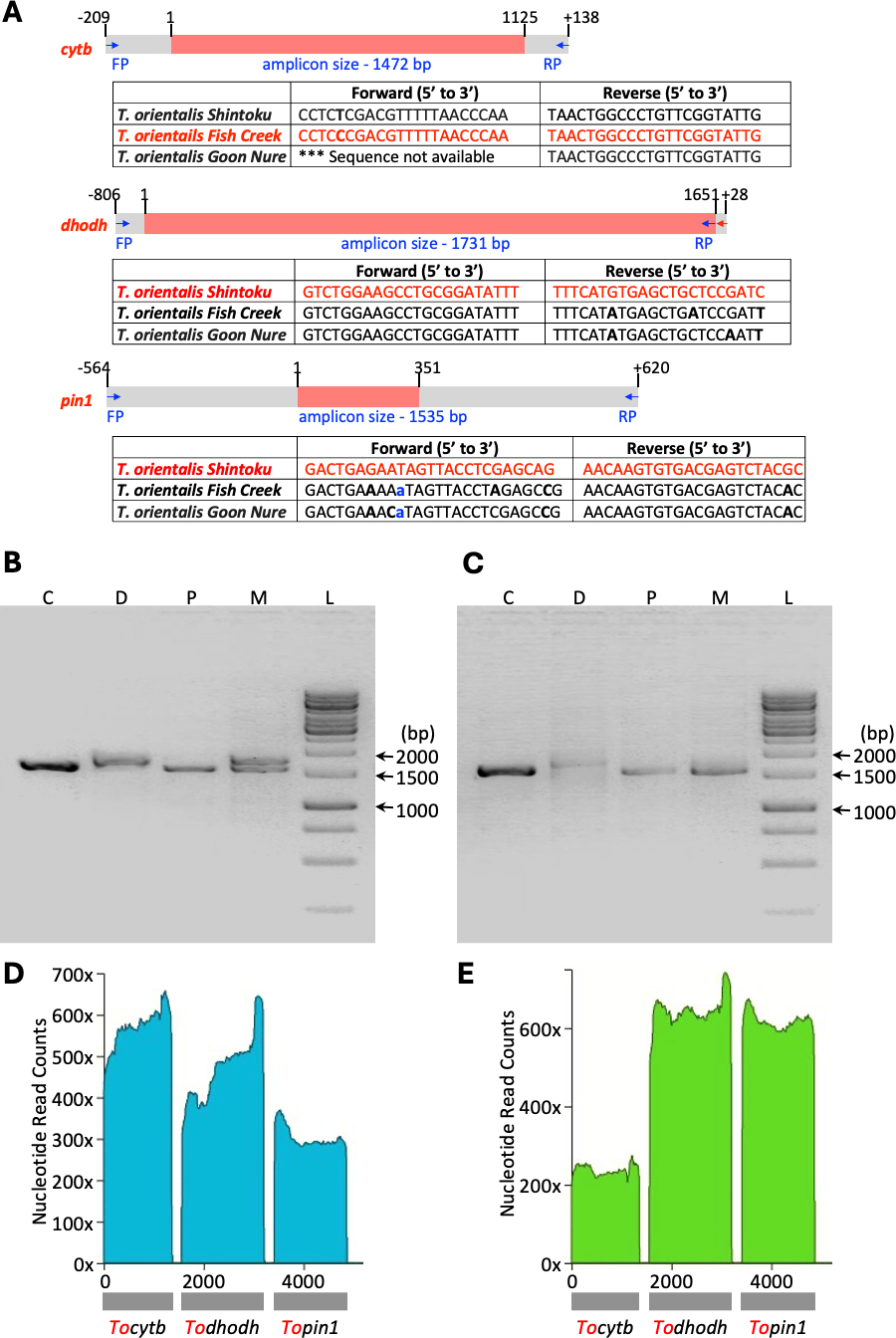

Supplement: S2 Fig — A, Schematic representation of coding region of T. orientalis cytb, dhodh, and pin1 genes (red colour) and flanking sequences (grey colour) is shown along with a table listing the PCR primers used in the study. Representative data for PCR amplification (B, C) and nanopore sequencing (D, E) of cytb (C), dhodh (D), pin1 (P) and multiplexed analysis of all three genes (M) from T. orientalis species. DNA templates used for PCR and sequencing were obtained from Kerala isolate (B, D) and a representative field sample (C, E). Lane L in B and C, 1 kb DNA marker ladder. Nucleotide-level mapping of nanopore sequence data for multiplexed PCR amplicons was plotted in real-time using the RAMPART program. (TIF) [file pone.0326243.s002.tif]

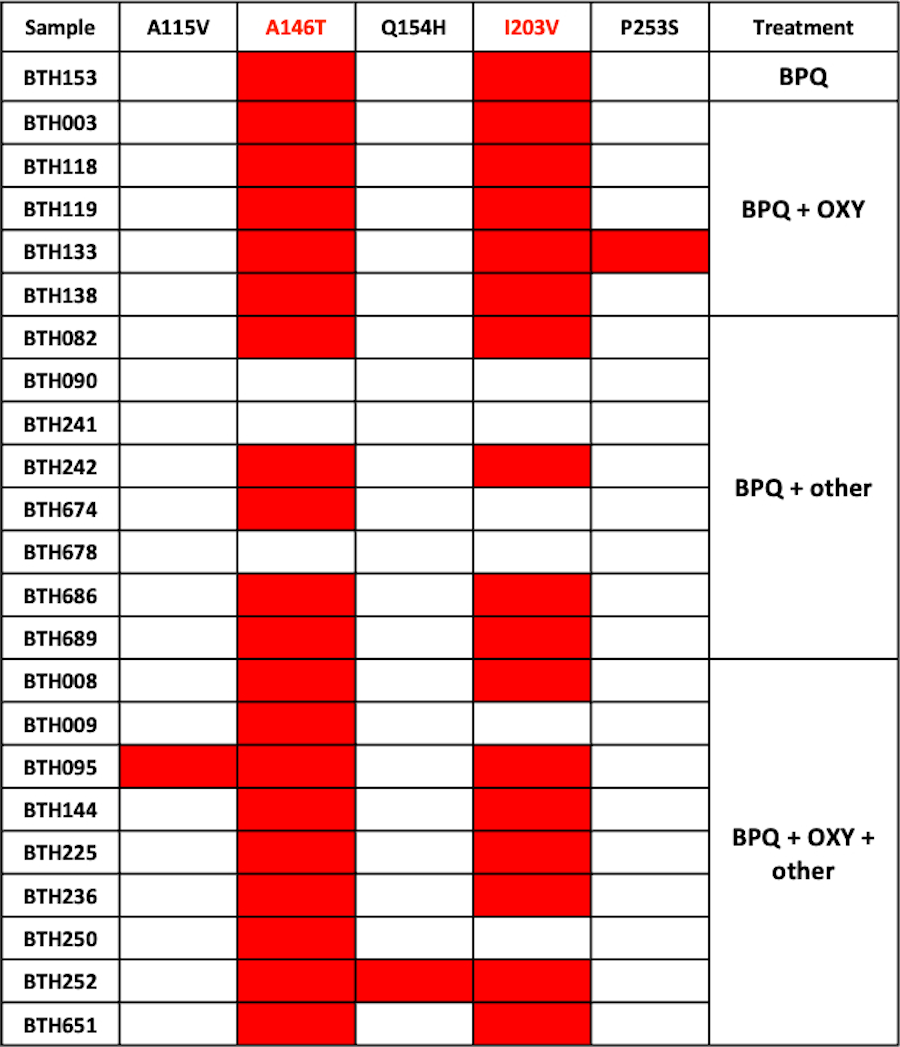

Supplement: S3 Fig — The genetic variations detected in the Tacytb gene from each sample is shown in red shading. The last column shows that drug treatment given for the individual animals. (TIF) [file pone.0326243.s003.tif]
